# Pathogenic Bacterium *Acinetobacter baumannii* Inhibits the Formation of Neutrophil Extracellular Traps by Suppressing Neutrophil Adhesion

**DOI:** 10.3389/fimmu.2018.00178

**Published:** 2018-02-07

**Authors:** Go Kamoshida, Takane Kikuchi-Ueda, Satoshi Nishida, Shigeru Tansho-Nagakawa, Tsuneyuki Ubagai, Yasuo Ono

**Affiliations:** ^1^Department of Microbiology and Immunology, School of Medicine, Teikyo University, Tokyo, Japan

**Keywords:** *Acinetobacter baumannii*, neutrophil, neutrophil extracellular trap, adhesion, CD11a

## Abstract

Hospital-acquired infections caused by *Acinetobacter baumannii* have become problematic because of high rates of drug resistance. *A. baumannii* is usually harmless, but it may cause infectious diseases in an immunocompromised host. Although neutrophils are the key players of the initial immune response against bacterial infection, their interactions with *A. baumannii* remain largely unknown. A new biological defense mechanism, termed neutrophil extracellular traps (NETs), has been attracting attention. NETs play a critical role in bacterial killing by bacterial trapping and inactivation. Many pathogenic bacteria have been reported to induce NET formation, while an inhibitory effect on NET formation is rarely reported. In the present study, to assess the inhibition of NET formation by *A. baumannii*, bacteria and human neutrophils were cocultured in the presence of phorbol 12-myristate 13-acetate (PMA), and NET formation was evaluated. NETs were rarely observed during the coculture despite neutrophil PMA stimulation. Furthermore, *A. baumannii* prolonged the lifespan of neutrophils by inhibiting NET formation. The inhibition of NET formation by other bacteria was also investigated. The inhibitory effect was only apparent with live *A. baumannii* cells. Finally, to elucidate the mechanism of this inhibition, neutrophil adhesion was examined. *A. baumannii* suppressed the adhesion ability of neutrophils, thereby inhibiting PMA-induced NET formation. This suppression of cell adhesion was partly due to suppression of the surface expression of CD11a in neutrophils. The current study constitutes the first report on the inhibition of NET formation by a pathogenic bacterium, *A. baumannii*, and prolonging the neutrophil lifespan. This novel pathogenicity to inhibit NET formation, thereby escaping host immune responses might contribute to a development of new treatment strategies for *A. baumannii* infections.

## Introduction

*Acinetobacter baumannii* is an aerobic gram-negative bacillus that is widely distributed in nature. Recently, hospital-acquired infections caused by *A. baumannii* have become a severe problem because of high rates of drug resistance. The incidence of multidrug-resistant *A. baumannii* (MDRA) has increased rapidly worldwide since the late 1990s. Although, to control and treat *A. baumannii* infections, many studies have evaluated drug-resistance mechanisms and drug usage, MDRA is difficult to manage and remains a critical issue globally. The number of global fatalities associated with *A. baumannii* infections continues to rise (1–5).

Bacterial capsule, biofilm formation, secretion systems, high adhesion capacity, and high-affinity iron acquisition have been reported as the virulence factors of *A. baumannii* (6–11). Although *A. baumannii* possesses multiple potential pathogenicity factors, specific factors contributing to its virulence remain unclear, and, hence, it is currently not possible to satisfactorily manage infections caused by this bacterium. *A. baumannii* is usually harmless but it may cause various infectious diseases in an immunocompromised host (1, 3, 5). Therefore, bacterial interaction with host cells should be investigated to understand diseases linked to *A. baumannii* infection. However, details of the interaction between *A. baumannii* and host cells remain to be elucidated.

Bacteremia and severe sepsis are caused by *A. baumannii* in an immunocompromised host with high frequency, and are associated with high mortality rates (12–14). In a previous study, we reported that *A. baumannii* adheres to neutrophils and enhances their invasive ability, with the bacteria transported together with the invading neutrophils. *A. baumannii* appears to spread throughout the body by attracting and hijacking neutrophils, similar to a commuter calling a taxi cab, and causing bacteremia. This novel mechanism of bacterial mobility is referred to as the “bacterial immunity taxi” (15).

Neutrophils are immune cells that play a pivotal role during the initial immune response to various bacterial infections (16). Recently, neutrophils were shown to release their nuclear content, including unfolded chromatin and lysosomal enzymes. This phenomenon plays a critical role in bacterial killing by trapping and inactivating the bacteria. This biological defense mechanism is termed neutrophil extracellular traps (NETs) (17–20). We have previously investigated neutrophil responsiveness to *A. baumannii* and *Pseudomonas aeruginosa* (a bacterium closely related to *A. baumannii*), focusing on NETs (21). We found that *A. baumannii* does not induce NET formation and cannot sterilize bacteria, in contrast to *P. aeruginosa*. Moreover, phagocytosis and the production of reactive oxygen species (ROS) were less pronounced in neutrophils stimulated by *A. baumannii* than by *P. aeruginosa*. Our previous studies suggest that *A. baumannii* is able to escape neutrophil defense mechanisms (15, 21).

Recently, it was paper reported that the probiotic bacterium *Lactobacillus rhamnosus* inhibits NET formation *via* its antioxidative activity (22). The notion that bacteria control NET formation, a defense mechanism of neutrophils against infection, is worth pursuing. Moreover, NETs are considered to be a type of cell death mechanism, termed NETosis (19, 23), and it would be very interesting if bacteria could suppress the death of immune cells. Many pathogenic bacteria have been reported to induce NET formation, while an inhibitory effect on NET formation is rarely reported (20, 24, 25). *A. baumannii* may possess a number of escape mechanisms enabling it to evade several immune responses, as demonstrated above. In the current study, we investigated the inhibitory effect of *A. baumannii* on NET formation. We found that *A. baumannii* inhibits NET formation *via* the action of phorbol 12-myristate 13-acetate (PMA)-induced neutrophils. Furthermore, we provide evidence that suppression of neutrophil adhesion underpins this phenomenon.

## Materials and Methods

### Bacteria and Neutrophils

*Acinetobacter baumannii* (ATCC 19606), *A. calcoaceticus* (ATCC 14987), *A. haemolyticus* (ATCC 17906), and *Escherichia coli* (ATCC 25922) were used as reference strains. Bacteria were grown in LB broth (Sigma-Aldrich, St. Louis, MO, USA) for 16 h at 37°C. The cells were then washed with phosphate-buffered saline (PBS), and suspended in fresh RPMI 1640 medium (Sigma-Aldrich).

Neutrophils were isolated from the human peripheral blood of healthy volunteers (*n* = 10), as described previously (26). Heparinized human blood was mixed with dextran 200,000/saline (final concentration of 1%) to sediment most of the erythrocytes. The supernatant was then processed by density gradient centrifugation using lymphosepar I (Wako Pure Chemical Industries, Osaka, Japan). Neutrophils were purified from the pelleted cells after hypotonic conditions induced lysis of the remaining erythrocytes. Purity of the neutrophil preparation was greater than 95%, as assessed by Diff Quick staining.

Written informed consent was obtained from all study participants, in accordance with the Declaration of Helsinki. The study was approved by the Ethical Review Committee of the School of Medicine of Teikyo University.

### NET Visualization

Neutrophils (1 × 10^6^ cells/mL) and *A. baumannii* cells [5 × 10^7^ colony-forming units, i.e., multiplicity of infection (MOI) of 50] were cocultured on non-coated glass slides (As One, Osaka, Japan) for 3 h in RPMI 1640 containing 2% human serum, at 37°C and under an atmosphere of 5% CO_2_. In some experiments, neutrophils were stimulated with 200-nM PMA simultaneously with the culture. The cells were fixed with 5% formaldehyde at room temperature for 10 min, and washed three times with PBS. DNA was stained with 4′,6-diamidino-2-phenylindole (DAPI; Southern Biotech, Birmingham, AL, USA) and visualized under a fluorescence microscope (BX53; Olympus, Tokyo, Japan).

### NET Quantification

Neutrophils (1 × 10^6^ cells/mL) and *A. baumannii* cells (MOI 50) were cocultured in 96-well culture plates (AGC Techno Glass, Shizuoka, Japan) for 0–5 h in RPMI 1640 containing 2% human serum, at 37°C and under an atmosphere of 5% CO_2_, and stimulated with 200-nM PMA. Extracellular DNA was stained with 5-µM SYTOX green (Life Technologies, Gaithersburg, MD, USA), a fluorescent membrane-impermeable DNA dye. Fluorescence was quantified using a microplate reader equipped with filters to detect the excitation and emission maxima at 485 and 535 nm, respectively (ARVO MX; PerkinElmer, Waltham, MA, USA). In some experiments, the assay was performed in the presence of 0.1-µg/mL CD11a-blocking antibody or control IgG (BioLegend, San Diego, CA, USA).

### Cell Cytotoxicity Assay

The release of lactate dehydrogenase (LDH) from neutrophils was detected by using an MTX-LDH assay kit (Kyokuto Pharmaceutical Industrial Co., Ltd., Tokyo, Japan). Briefly, LDH reduces NAD to NADH, leading to the production of formazan in proportion to the LDH levels in cell supernatants; the formazan produced was determined at 560 nm in a microplate reader. Cytotoxicity was calculated as a percentage compared with LDH levels of lysed neutrophils.

### Neutrophil Survival Analysis

Neutrophils (1 × 10^6^ cells/mL) were cultured for 3 h in RPMI 1640 medium supplemented with 2% human serum, and viable neutrophils, which were not stained with trypan blue, were counted. In some experiments, neutrophils were stimulated with 200-nM PMA and cocultured with *A. baumannii* (MOI 50).

### Time-Lapse Observations

Neutrophils (2.5 × 10^5^ cells/0.25 mL), fluorescently labeled using PKH 26 (Sigma-Aldrich), were cultured in RPMI 1640 supplemented with 2% human serum, in the presence or absence of *A. baumannii* (MOI 50), and stimulated with 200-nM PMA. The cells were cultured in a glass-bottom dish (CELLview, Greiner Bio-One, Frickenhausen, Germany) at 37°C under an atmosphere of 5% CO_2_. Extracellular DNA was visualized by staining with 5-µM SYTOX green. The cells were evaluated every minute by confocal microscopy, for 5 h (FV10i, Olympus).

### Cell-Adhesion Assay

Neutrophils (1 × 10^6^ cells/mL, 0.1 mL) were placed in a 96-well culture plate, and incubated at 37°C for 30 min in RPMI 1640 supplemented with 2% human serum. In some experiments, neutrophils were stimulated with 200-nM PMA when cocultured with *A. baumannii* (MOI 50), before adding them to the wells. The non-adhering neutrophils were removed by washing the wells three times with PBS. The adherent cells remaining in the wells were lysed with 1% Nonidet P-40, and LDH from adherent neutrophils was detected using an MTX-LDH assay kit, as described above. The adhesion rate of neutrophils was calculated as a percentage of the lysed neutrophils. In some experiments, the assay was performed in the presence of 0.1-µg/mL CD11a-blocking antibody or control IgG.

### Flow-Cytometry Analysis

The surface expression of CD11a and CD11b on neutrophils was determined by flow cytometry (FACSCanto II, BD Biosciences, San Jose, CA, USA), using specific antibodies [FITC-labeled anti-CD11a antibody and PE-labeled anti-CD11b antibody (BD Biosciences)].

### Statistical Analysis

Data are presented as the mean ± SD. The values were compared using paired Student’s *t*-test, and differences at *p* < 0.01 were considered statistically significant.

## Results

### *A. baumannii* Inhibiting NET Formation

**Figure 1:**
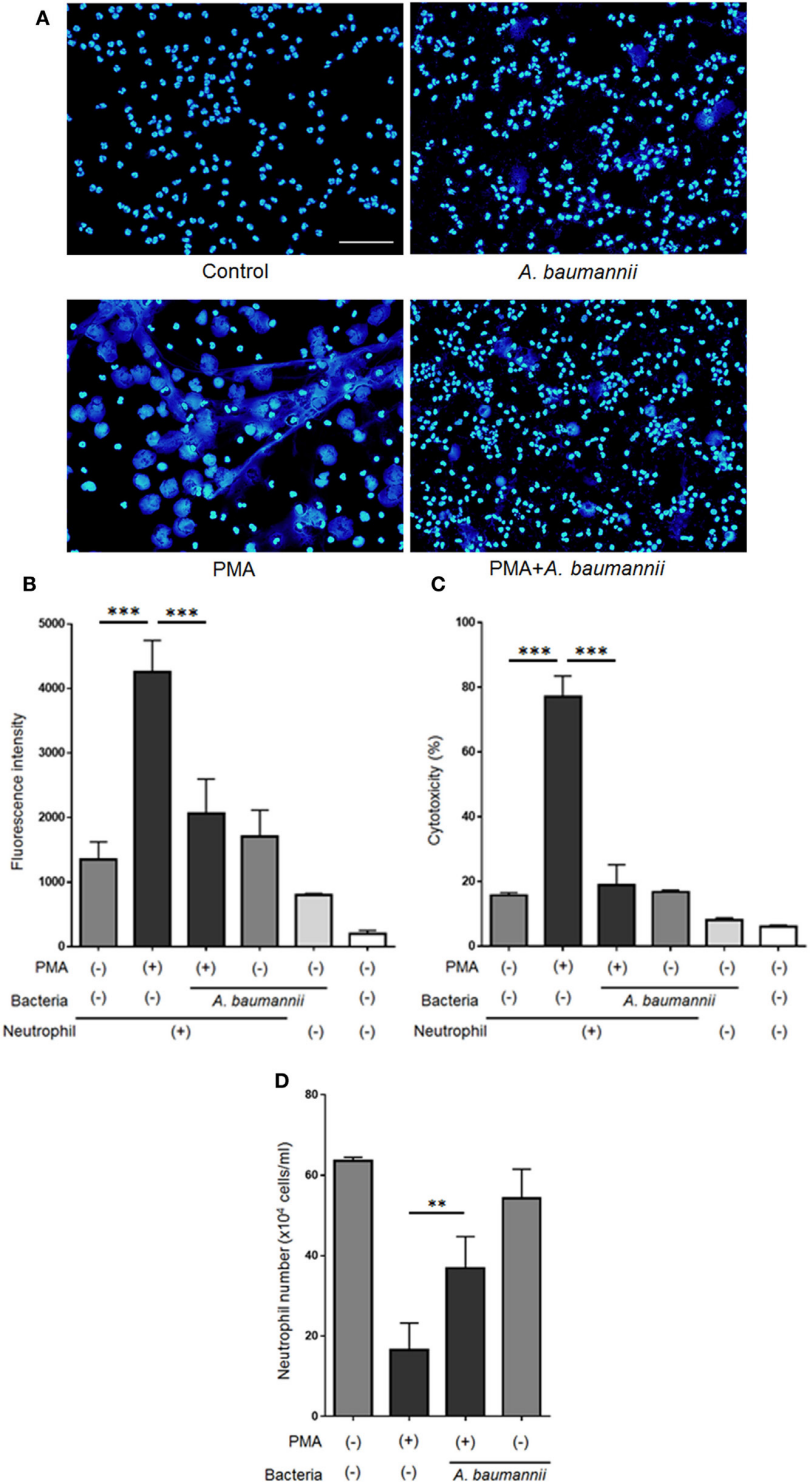
*Acinetobacter baumannii* inhibiting the formation of NETs. **(A)** Neutrophils and *A. baumannii* (MOI 50) were cocultured for 3 h, in the presence or absence of 200-nM phorbol 12-myristate 13-acetate (PMA). After incubation, the cells were fixed and DNA were stained with DAPI. Scale bar, 50 µm. **(B)** Extracellular DNA signal, after staining with SYTOX green and quantification. **(C)** Levels of extracellular lactate dehydrogenase (LDH) released by neutrophils, determined by the MTX-LDH assay kit. Cytotoxicity was calculated as a percentage based on LDH levels of lysed neutrophils. **(D)** Cell survival, based on staining with trypan blue. The data are shown as the mean ± SD; *n* ≥ 3 per group. ***p < 0.001 and **p < 0.01. The results are representative of at least three experiments.

Previously, we reported that *A. baumannii* does not induce NET formation after a 1-h coculture with neutrophils and that neutrophils are unable to kill this bacterium (21). Thus, *A. baumannii* appeared to possess escape mechanisms to evade immune responses. In the present study, we investigated the effect of *A. baumannii* on PMA-induced NET formation. Neutrophils and *A. baumannii* were cocultured for 3 h in the presence of PMA, DNA was stained with DAPI, and observed under a fluorescence microscope. NET formation was observed upon PMA stimulation. However, NETs were rarely observed when neutrophils were cocultured with *A. baumannii*, regardless of PMA stimulation (Figure 1A). To quantify the amount of the released extracellular DNA, the signal of SYTOX green-stained DNA in the medium was measured. The amount of extracellular neutrophil DNA increased after PMA stimulation, but it was significantly reduced upon coculture with *A. baumannii* (Figure 1B). Furthermore, neutrophil cell death was evaluated by determining the amount of released LDH. Extracellular LDH levels were elevated upon PMA stimulation, but, in comparison, they were significantly reduced upon coculture with *A. baumannii* (Figure 1C). Next, the survival of PMA-stimulated neutrophils was evaluated in coculture with *A. baumannii*. Upon PMA stimulation, most neutrophils died; in contrast, many remained alive during coculture with *A. baumannii* (Figure 1D). Furthermore, *A. baumannii* alone did not induce neutrophil cell death. Consequently, *A. baumannii* prolonged the lifespan of neutrophils by inhibiting NET formation, i.e., a cell death pathway, in PMA stimulation.

**Figure 2:**
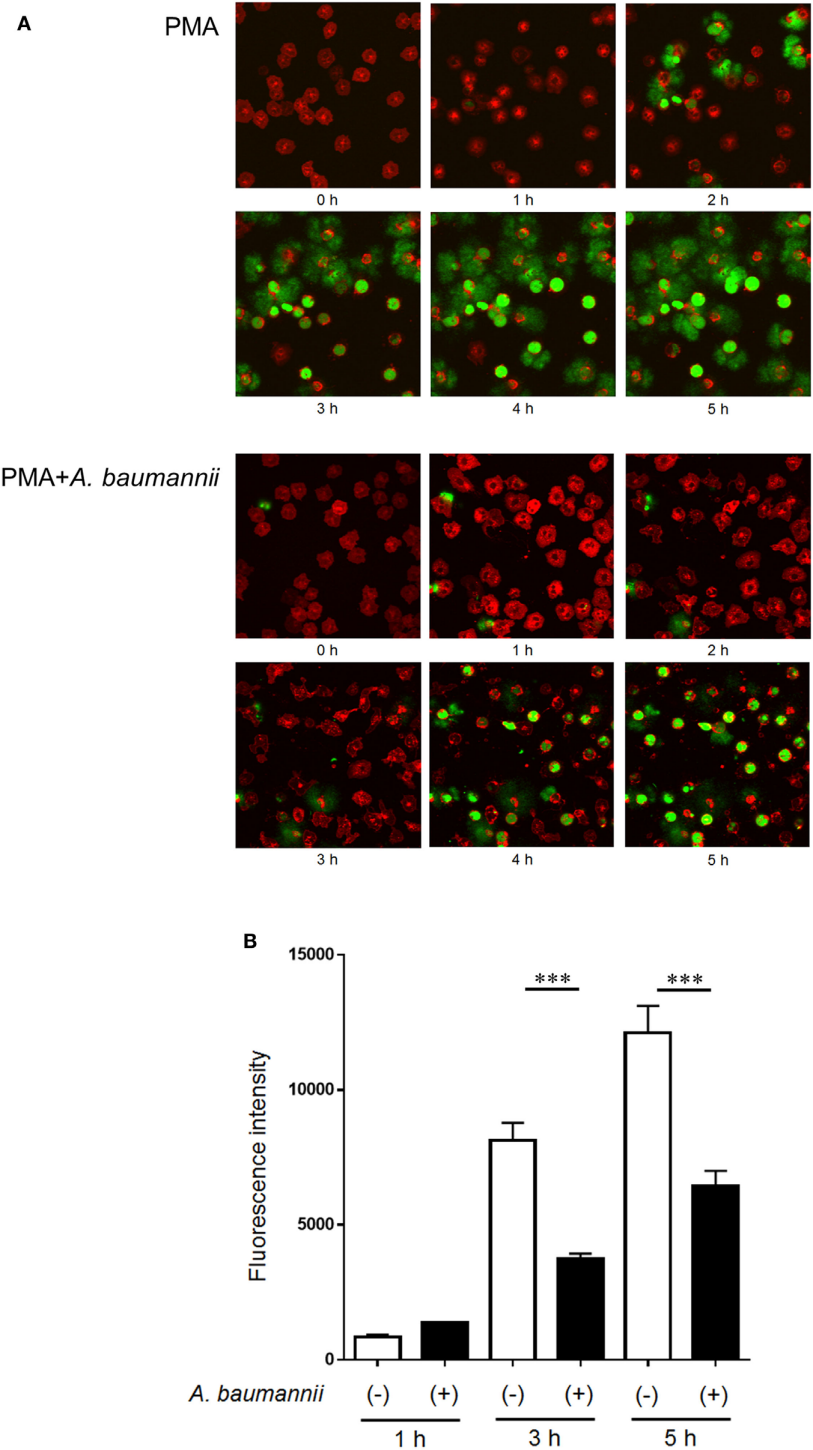
Kinetics of the inhibition of NET formation by *Acinetobacter baumannii*. **(A)** Fluorescently labeled neutrophils (red) and *A. baumannii* (MOI 50) were cocultured for 0–5 h in the presence of 200-nM phorbol 12-myristate 13-acetate (PMA); SYTOX green (green) was used to visualize the extracellular DNA. Cells were observed every minute by confocal microscopy. Hourly images are shown. **(B)** Quantification of DNA released by neutrophils during the experiment described in **(A)**. The data are shown as the mean ± SD; *n* ≥ 3 per group. ***p < 0.001. The results are representative of at least three experiments.

Furthermore, to investigate the kinetics of the inhibitory effect of *A. baumannii* on NET formation, NET formation was monitored during a 5-h neutrophil culture with PMA, in the presence or absence of *A. baumannii* by time-lapse microscopy (Figure 2A; Videos S1 and S2 in Supplementary Material), and detection of extracellular DNA (Figure 2B). As observed, PMA-induced NET formation was inhibited by coculture with *A. baumannii* for up to 5 h. Compared with PMA stimulation alone, the amount of extracellular neutrophil DNA was also inhibited by coculture with *A. baumannii*. Hence, *A. baumannii* inhibited PMA-induced NET formation, thereby inhibiting PMA-stimulated cell death.

### Inhibitory Effect of Various Bacteria on NET Formation

**Figure 3:**
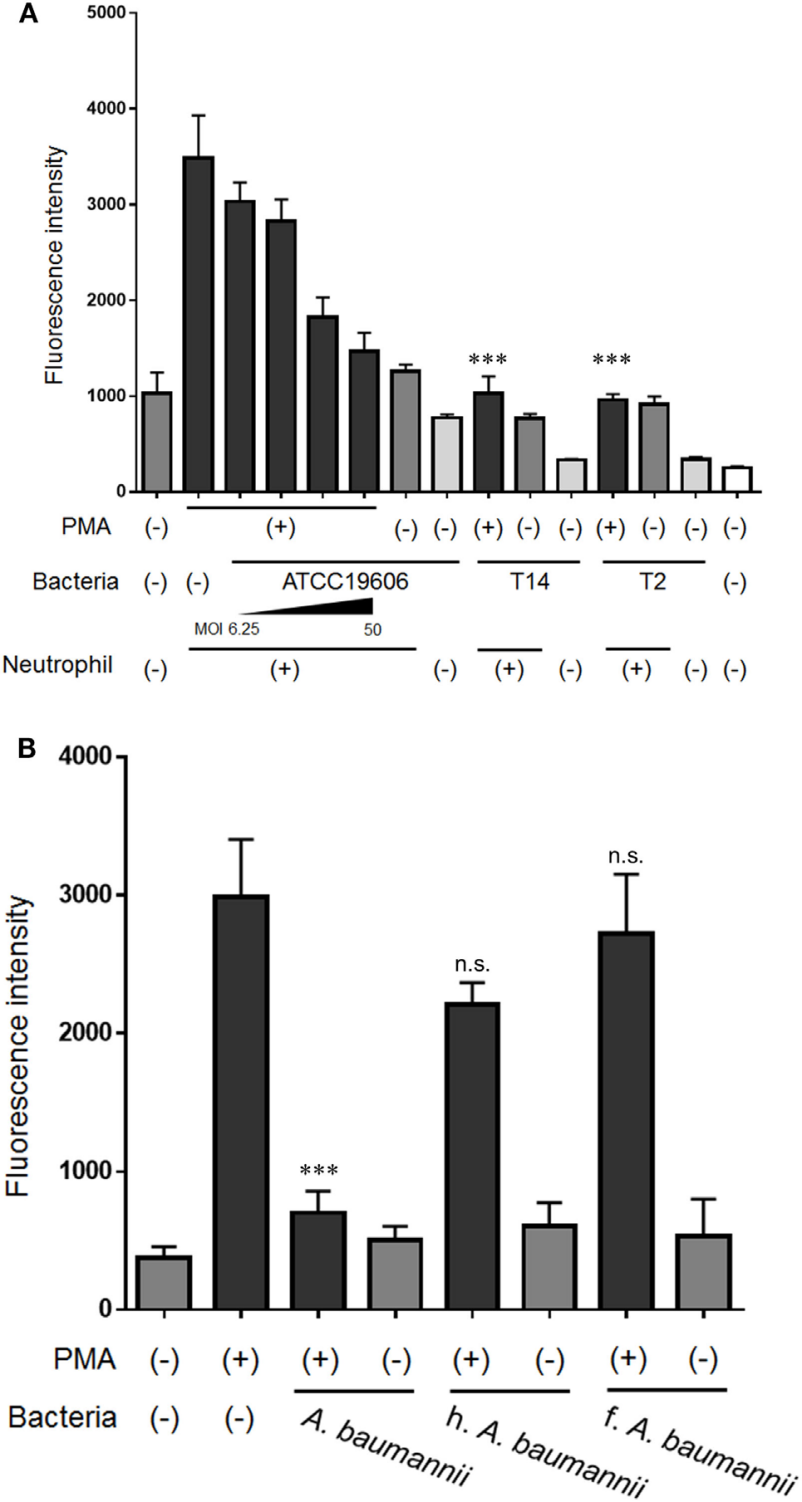
Inhibition of NET formation by *Acinetobacter baumannii* under different experimental conditions. **(A)** Neutrophils and ATCC 19606 (*A. baumannii* reference strain; MOI 6.25, 12.5, 25, and 50), or T14 or T2 (MDRA clinical isolates; MOI 50) were cocultured for 3 h, in the presence or absence of 200-nM phorbol 12-myristate 13-acetate (PMA). Extracellular DNA was stained with SYTOX green and the signal quantified. **(B)** NET formation in the presence of dead *A. baumannii* cells (MOI 50). The bacteria were either heat-killed (h.) or formalin-killed (f.). The data are shown as the mean ± SD; *n* ≥ 3 per group; n.s., not significant; ***p < 0.001 relative to PMA stimulation in the absence of bacteria. The results are representative of at least three experiments.

To investigate the inhibitory effect of *A. baumannii* on NET formation in more detail, the formation of NETs was quantified in the presence of various bacteria. *A. baumannii* reference strain (ATCC 19606) suppressed PMA-induced NET formation in an MOI-dependent manner. MDRA clinical isolates (T14 and T2) exerted the same inhibitory effect (Figure 3A). In another experiment, *A. baumannii* cells were heat-killed or formalin-killed, and the NET quantification assay was performed. Upon killing, the bacteria lost the ability to inhibit NET formation (Figure 3B).

**Figure 4:**
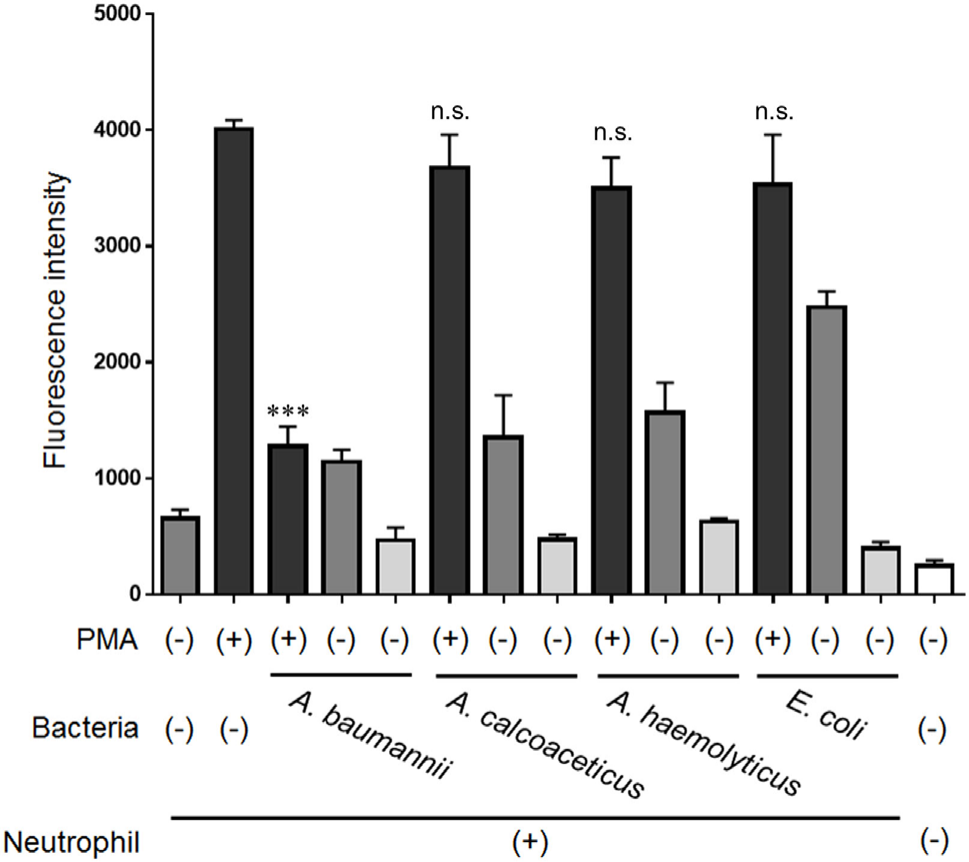
Inhibition of NET formation by bacteria other than *Acinetobacter baumannii*. Neutrophils and *A. baumannii, A. calcoaceticus, A. hemolyticus*, or *Escherichia coli* (MOI 50) were cocultured for 3 h, in the presence or absence of 200-nM phorbol 12-myristate 13-acetate (PMA). Extracellular DNA was stained with SYTOX green and the signal quantified. The data are shown as the mean ± SD; *n* ≥ 3 per group; n.s., not significant; ***p < 0.001 relative to PMA stimulation in the absence of bacteria. The results are representative of at least three experiments.

Next, the ability of bacteria other than *A. baumannii* to inhibit NET formation was examined. Other bacteria from the same genus, *A. calcoaceticus* and *A. hemolyticus*, and bacteria from a different genus, *E. coli*, did not inhibit NET formation (Figure 4). These observations suggested that only live *A. baumannii* cells are able to inhibit PMA-induced NET formation.

### *A. baumannii* Inhibiting NET Formation by Suppressing Neutrophil Adhesion

**Figure 5:**
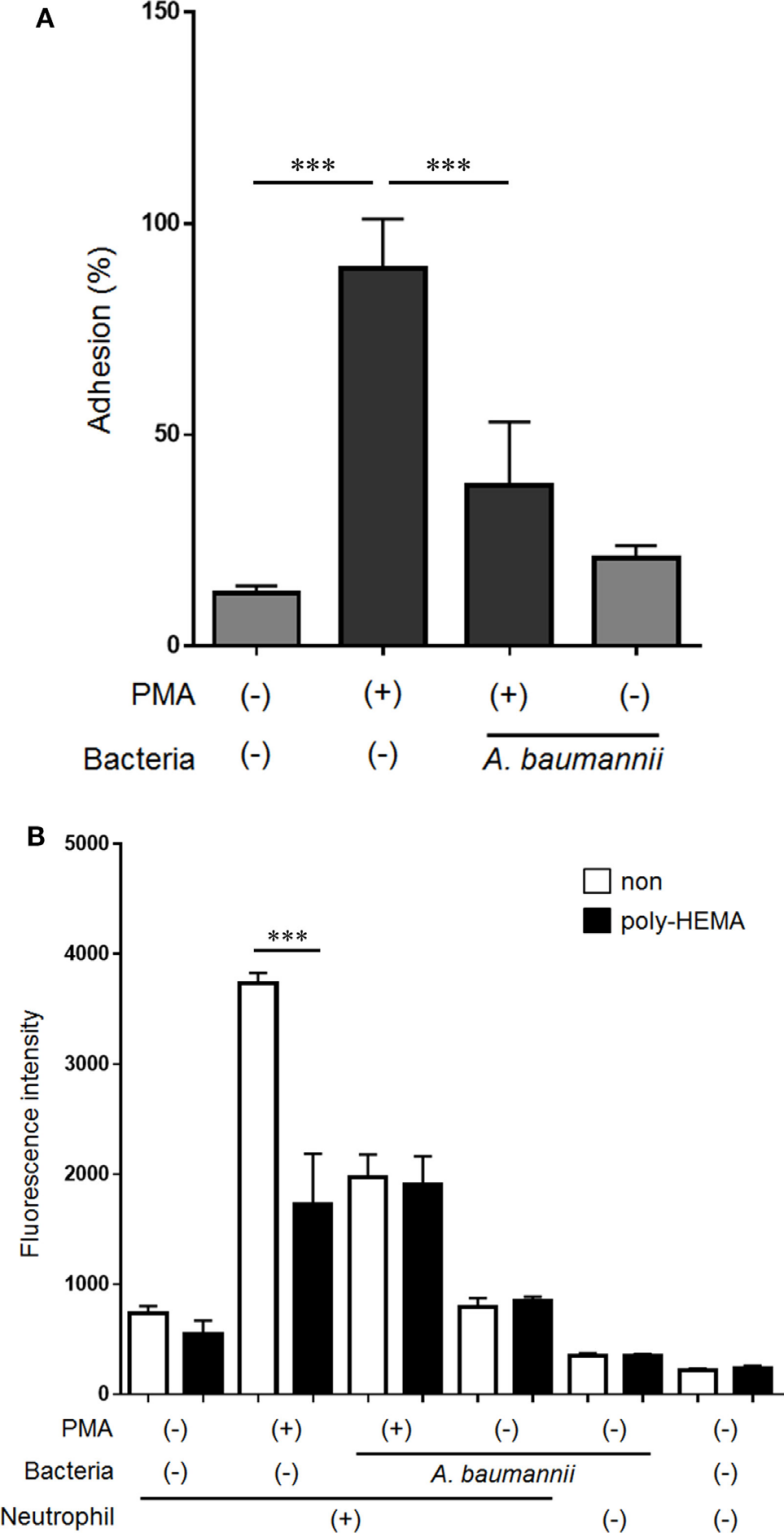
*Acinetobacter baumannii* inhibiting NET formation by suppressing neutrophil adhesion. **(A)** Neutrophils were stimulated with 200-nM phorbol 12-myristate 13-acetate (PMA) and cocultured with *A. baumannii* (MOI 50) for 30 min. The non-adhering neutrophils were removed, the adhering cells were lysed, and LDH levels determined. The adhesion rate was calculated as a percentage compared with lysed neutrophils. **(B)** Neutrophils were cultured for 3 h in poly-HEMA-coated plates (poly-HEMA inhibits cell adhesion), in the absence or presence of 200-nM PMA, and *A. baumannii* (MOI 50). Extracellular DNA was stained with SYTOX green and the signal quantified. The data are shown as the mean ± SD; *n* ≥ 3 per group. ***p < 0.001. The results are representative of at least three experiments.

Neutrophil adhesion to the scaffold is thought to be required for NET formation. However, we previously demonstrated that neutrophil mobility is enhanced upon stimulation with *A. baumannii* (15). Furthermore, we evaluated neutrophil morphology at 1 h post PMA stimulation. The cytoplasm in PMA-stimulated neutrophils was thin and roundly spread, and these neutrophils appeared to adhere strongly. In contrast, PMA and *A. baumannii*-stimulated neutrophils appeared to adhere weakly compared with only PMA-stimulated neutrophils (Figure S1 in Supplementary Material). Therefore, we speculated that the adhesion ability of neutrophils was reduced upon *A. baumannii* stimulation. Cell-adhesion assay was performed to evaluate the adhesion ability of neutrophils upon PMA stimulation and coculture with *A. baumannii*. The adhesion ability was enhanced by PMA stimulation, but this enhancement was suppressed during coculture with *A. baumannii* (Figure 5A). Next, NET quantification assay was performed using plates coated with a cell-adhesion inhibitor, poly-2-hydroxyethyl methacrylate (poly-HEMA), to examine whether inhibition of adhesion would affect NET formation. Indeed, PMA-induced NET formation was suppressed to the *A. baumannii* coculture levels when the neutrophil adhesion was inhibited (Figure 5B). These observations suggested that the inhibition of neutrophil adhesion suppresses NET formation. Furthermore, we found that *A. baumannii* could suppress the adhesion of PMA-stimulated neutrophils.

### *A. baumannii* Inhibiting NET Formation by Inhibiting Neutrophil CD11a Expression

**Figure 6:**
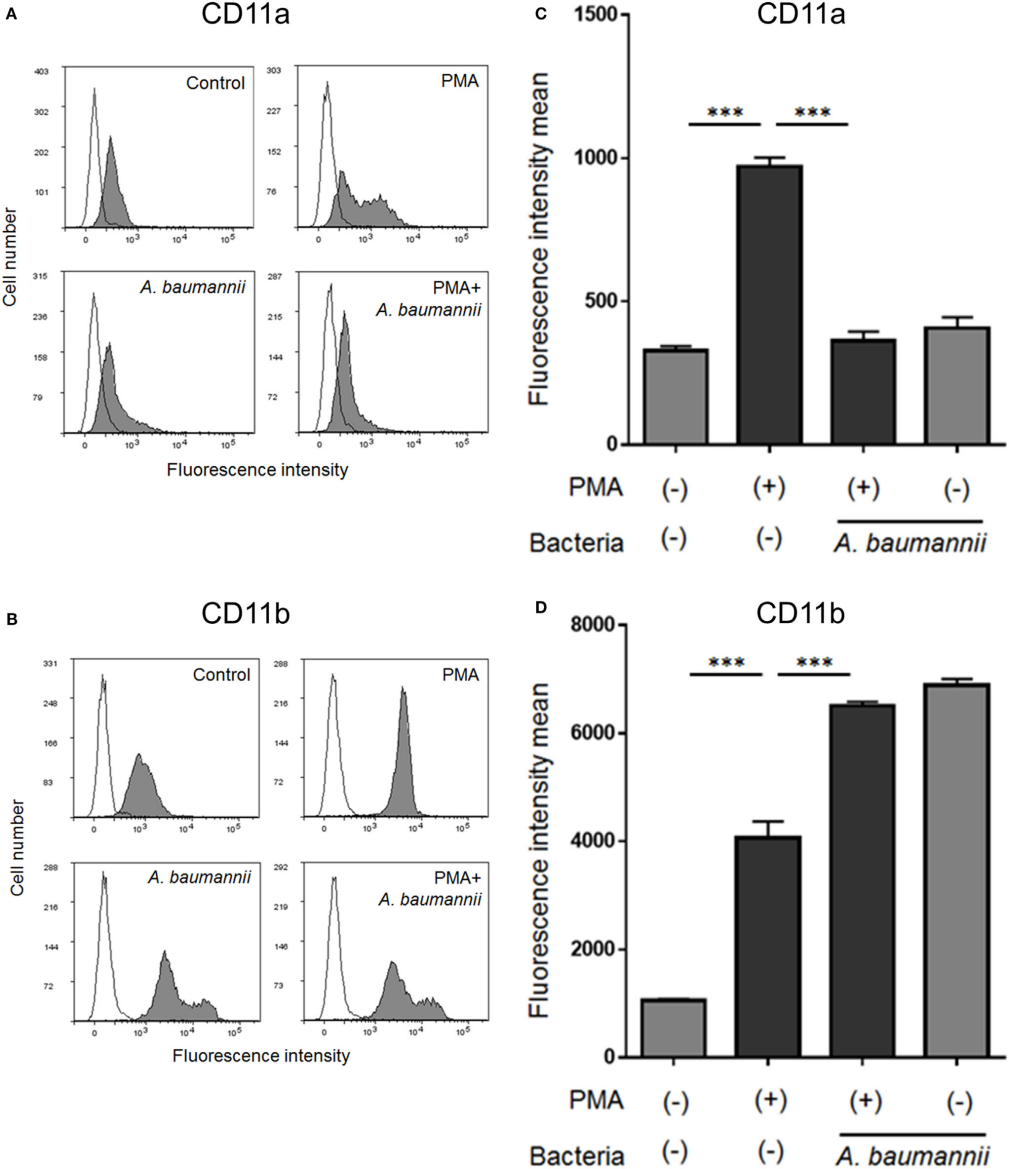
Changes in the cell surface expression of neutrophil molecules CD11a and CD11b. Neutrophils were stimulated with 200-nM phorbol 12-myristate 13-acetate (PMA) and cocultured with *Acinetobacter baumannii* (MOI 50) for 1 h. The expression of CD11a and CD11b was determined by flow cytometry using specific antibodies. Representative histograms are shown. Fluorescence profiles in the absence of antibody staining are indicated by unfilled shapes **(A,B)**. The determined mean fluorescence intensity of samples is shown in **(C,D)**. The data are shown as the mean ± SD; *n* ≥ 3 per group. ***p < 0.001. The results are representative of at least three experiments.

Finally, to investigate the mechanism underlying the suppression of neutrophil adhesion upon stimulation with *A. baumannii*, flow cytometry was used to analyze the expression of neutrophil cell surface adhesion molecules. The levels of CD11a (integrin αL) and CD11b (integrin αM) were increased upon PMA stimulation. The enhancement of CD11a expression by PMA was suppressed upon coculture with *A. baumannii* (Figures 6A,C). In contrast, the enhancement of CD11b expression was further increased in the presence of *A. baumannii* (Figures 6B,D).

**Figure 7:**
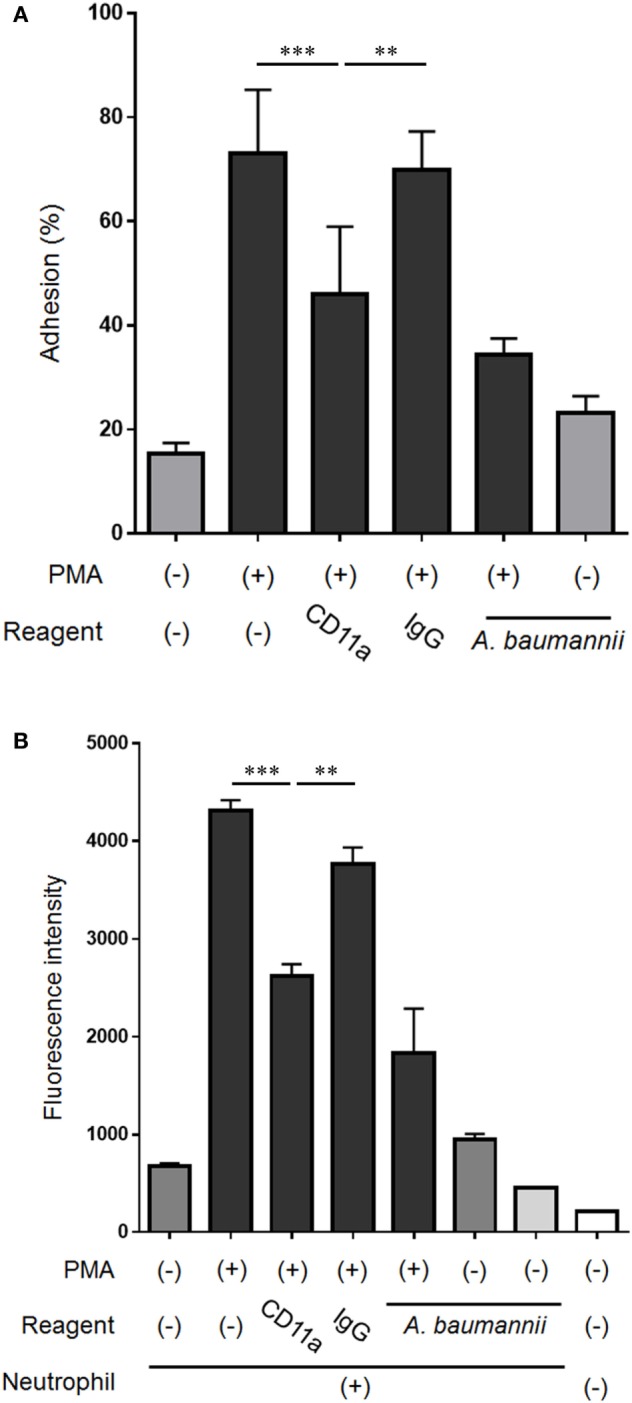
Effect of the neutrophil cell surface molecule CD11a on NET formation. **(A)** Neutrophils were stimulated with 200-nM phorbol 12-myristate 13-acetate (PMA) for 30 min, the non-adherent neutrophils were removed, the adhering cells were lysed, and LDH levels were determined. In some experiments, the assay was performed in the presence of 0.1-µg/mL CD11a-blocking antibody or control IgG, and *Acinetobacter baumannii* (MOI 50). The adhesion rate was calculated as a percentage compared with lysed neutrophils. **(B)** Neutrophils were cultured for 3 h, and the extracellular DNA was stained with SYTOX green and the signal quantified. Some experiments were performed in the presence of 0.1-µg/mL CD11a-blocking antibody or control IgG, and *A. baumannii* (MOI 50). The data are shown as the mean ± SD; *n* ≥ 3 per group. ***p < 0.001 and **p < 0.01. The results are representative of at least three experiments.

Because the expression of CD11a in neutrophils was suppressed by *A. baumannii*, the effect of CD11a on the neutrophil adhesion ability and NET formation was next assessed. The enhancement of neutrophil adhesion by PMA was reduced in the presence of a CD11a-blocking antibody (Figure 7A). Furthermore, PMA-induced NET formation was also reduced when a CD11a-blocking antibody was present (Figure 7B). Collectively, these observations suggested that *A. baumannii* suppressed the neutrophil expression of CD11a, thereby inhibiting PMA-induced NET formation.

## Discussion

A previous study reported that the probiotic bacterium *L. rhamnosus* inhibits NET formation (22). The immunomodulatory effect of probiotic bacteria, including lactic acid bacteria such as *L. rhamnosus*, on host immune cells is well known (27). Furthermore, it was reported that sugar constituents of the *Cryptococcus neoformans* capsule and of the *Candida albicans* or *Streptococcus suis* biofilms modulate NET formation (28–30). However, many common pathogenic bacteria were reported to induce NET formation, while the inhibition of NET formation is rarely reported (20, 24, 25). The current study constitutes the first-ever report that *A. baumannii*, a common pathogenic gram-negative bacterium, exerts such an inhibitory effect. Furthermore, it is surprising that the pathogenic bacterium *A. baumannii* prolongs the lifespan of neutrophils by inhibiting NET formation. Previous studies reported that *L. rhamnosus* and microbial sugars suppress ROS production in neutrophils, thereby inhibiting NET formation. Therefore, ROS production by *A. baumannii*-stimulated neutrophils was also investigated. The enhancement of ROS production by PMA was not suppressed by *A. baumannii* stimulation (Figure S2 in Supplementary Material). Thus, *A. baumannii* did not appear to possess an antioxidative activity. Moreover, no capsule or biofilm formation by *A. baumannii* was observed under the culture conditions employed in the current study. Furthermore, *L. rhamnosus* was shown to inhibit bacterium-induced NET formation (22). The effect of *A. baumannii* on *E. coli*-induced NET formation was hence investigated. However, *A. baumannii* did not inhibit this process (Figure S3 in Supplementary Material). Various pathways of NET formation are known (25, 31, 32). Based on data presented in the current study, we suggest that *A. baumannii* inhibits the formation of NETs that require long (3 h or more) and strong neutrophil activation, e.g., PMA stimulation, independently of antioxidative activity.

Cell adhesion is important for neutrophil activation (16, 33). We thought that NETs are also similar, with the neutrophils unable to release their DNA into the extracellular space without adhesion, but this was clearly not the case. In the current study, we showed that PMA-induced neutrophil NET formation was suppressed when the assay was performed using plates treated with a cell-adhesion inhibitor. Furthermore, we showed that *A. baumannii* inhibits neutrophil adhesion by suppressing the PMA-induced CD11a expression, thereby inhibiting NET formation. However, CD11a inhibition did not completely suppress the enhancement in adhesion stimulated by PMA. CD11b inhibition also suppressed adhesion and NET formation of neutrophils induced by PMA (Figure S4 in Supplementary Material). Thus, cell adhesion is important for NET formation, and other cell-adhesion molecules may also be partially involved in this effect. In the current study, we demonstrated that CD11a is an important player in neutrophil adhesion and NET formation after PMA stimulation, and its levels are modulated by *A. baumannii* stimulation.

Many neutrophil surface molecules also change upon stimulation and regulate neutrophil activity (34, 35). Recently, Zawrotniak et al. reported neutrophil surface molecules and signals that are important for promoting NET formation stimulated by *C. albicans* components such as mannan (36). Also, glucuronoxylomannan from the *C. neoformans* capsule (28) and matrix mannan from the *C. albicans* biofilm (29) inhibit NET formation. Thus, NET formation appears to be regulated *via* a complex mechanism involving the microorganism component and its neutrophil surface receptors. Indeed, in this study, CD11b, CD14, CD16, TLR4, and TLR2 expression was also affected by PMA stimulation and *A. baumannii* cocultivation (Figures 6B,D and Figure S5 in Supplementary Material). Investigating the bacterial factor(s), associated receptor(s), and signals is thus a challenge for future studies.

*Acinetobacter baumannii* appears to inhibit the activation of neutrophils by suppressing their adhesion, and extends their survival after PMA stimulation. Furthermore, when the neutrophils were cocultured with *A. baumannii*, the extracellular LDH levels were low and the majority of neutrophils remained alive. In contrast, many neutrophils died upon coculture with *E. coli* or *P. aeruginosa* (Figure S6 and Video S3 in Supplementary Material). Although it is generally known that many common bacteria kill neutrophils by inducing apoptosis or exerting cytotoxicity (37, 38), *A. baumannii* did not exert such a killing effect. Furthermore, our previous studies suggested that *A. baumannii* avoids neutrophil defense mechanisms, such as phagocytosis, at an initial point (at 1 h) (21). It appears that *A. baumannii* is not recognized as a foreign body that stimulates a strong inflammatory response by neutrophils. However, this is clearly in contrast to the findings of other (39). Lazaro-Diez et al. showed that *A. baumannii* is indeed phagocytosed by neutrophils and induces NET formation. These discrepancies may be associated with the difference in the experimental conditions, such the duration of coculture, MOI, and culture conditions. In another report, macrophages rather than neutrophils were shown to contribute to phagocytosis during the initial stages of *A. baumannii* infection (40).

Neutrophils are key immune cells indispensable for the control of *A. baumannii* infection. Several studies reported the increase of mortality associated with *A. baumannii* infection upon neutrophil depletion in mouse models (41–43). The recruitment of neutrophils to the *A. baumannii* infection site *via* cytokines and chemokines has been evidenced in many studies, including our own (15, 41, 44). Immune strategies to combat *A. baumannii* may involve the triggering of oxidative burst and cytokine/chemokine production to amplify the immune response against the pathogen by the accumulated neutrophils (45). However, a part of *A. baumannii* can escape the immune response, including NETs, as demonstrated in the current study. In this case, *A. baumannii* would contribute to the dissemination of bacteria by utilizing the migratory capacity of neutrophils, as reported previously (15). Indeed, bacteremia and severe sepsis are caused by *A. baumannii* with high frequency in a compromised host (12–14). Furthermore, the bacteria are also detected in other organs, including the blood, in several mouse models of respiratory *A. baumannii* infection (42). The data reported in the current study suggest that *A. baumannii* inhibits NET formation, thereby escaping host immune responses and causing infection. This novel mechanism to inhibit NET formation might contribute to a development of new treatment strategies for *A. baumannii* infections.

In summary, we discovered that neutrophil adhesion is important for PMA-induced NET formation and that *A. baumannii* inhibits NET formation by suppressing neutrophil cell adhesion. Future studies should investigate the involved molecule(s) in detail and the mechanisms that control the expression of neutrophil CD11a, including bacterial factor(s) and intracellular signals.

## Ethics Statement

Written informed consent was obtained from all study participants, in accordance with the Declaration of Helsinki. The study was approved by the Ethical Review Committee of the School of Medicine of Teikyo University.

## Author Contributions

GK designed and performed the experiments and analyzed the data. TK-U, SN, ST-N, and TU performed some of the experiments and participated in data interpretation. YO supervised the study. GK and YO cowrote the manuscript.

## Conflict of Interest Statement

The authors declare that the research was conducted in the absence of any commercial or financial relationships that could be construed as a potential conflict of interest.

## Supplementary Material

Video S1 Time-lapse observation of PMA-induced NET formation. Fluorescently-labeled neutrophils (red) were cultured for 0–5 h in the presence of 200 nM PMA; SYTOX green (green) was used to visualize the extracellular DNA. The images were captured every minute by a confocal microscope.

Video S2 Time-lapse observation of the inhibition of PMA-induced NET formation by A. baumannii. Fluorescently-labeled neutrophils (red) and A. baumannii (MOI 50) were co-cultured for 0–5 h in the presence of 200 nM PMA; SYTOX green (green) was used to visualize the extracellular DNA. The images were captured every minute by a confocal microscope.

Video S3 Time-lapse observation of neutrophils co-cultured with A. baumannii. Fluorescently-labeled neutrophils (red) and A. baumannii (MOI 50) were co-cultured for 0–5 h; SYTOX green (green) was used to visualize the extracellular DNA. The images were captured every minute by a confocal microscope.

## Abbreviations

HEMA, 2-hydroxyethyl methacrylate; LDH, lactate dehydrogenase; MDRA, multidrug-resistant *Acinetobacter baumannii*; MOI, multiplicity of infection; NET, neutrophil extracellular trap; PMA, phorbol 12-myristate 13-acetate; ROS, reactive oxygen species.
