# Supplementary material for: Pathogenic Bacterium Acinetobacter baumannii Inhibits the Formation of Neutrophil Extracellular Traps by Suppressing Neutrophil Adhesion
Source: Front Immunol. 2018 Feb 7;9:178. doi: 10.3389/fimmu.2018.00178 (PMC5808340; doi:10.3389/fimmu.2018.00178)
Supplement: Supplementary file 4 [file Presentation_1.PDF]

Supplementary material

Pathogenic Bacterium *Acinetobacter baumannii* Inhibits the Formation of Neutrophil Extracellular Traps by Suppressing Neutrophil Adhesion

Go Kamoshida*, Takane Kikuchi-Ueda, Satoshi Nishida, Shigeru Tansho-Nagakawa, Tsuneyuki Ubagai, and Yasuo Ono

***Correspondence:** Go Kamoshida: kamoshida@med.teikyo-u.ac.jp

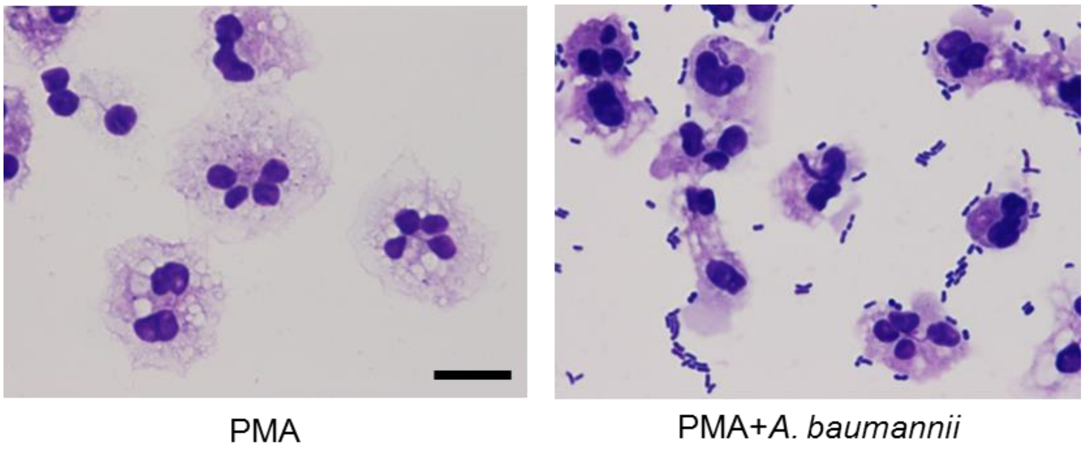

FIGURE S1 | Neutrophil adhesion morphology after PMA stimulation. Neutrophils were stimulated with 200 nM PMA and co-cultured with *A. baumannii* (MOI 50) for 1 h. These cells were then fixed and stained with Diff-Quik. Scale bar = 10 μm.

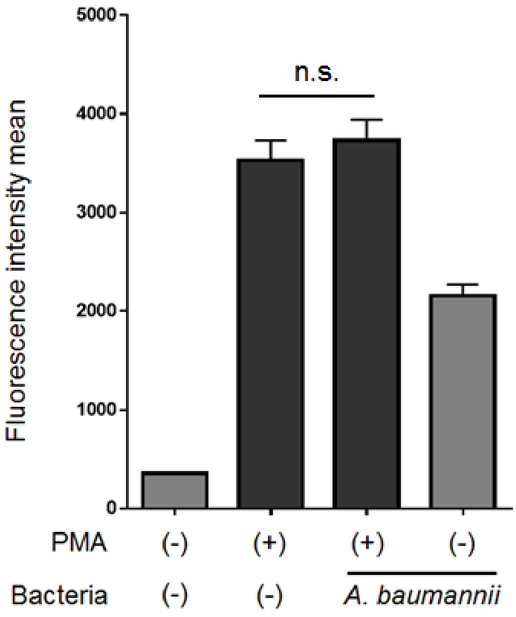

FIGURE S2 | *A. baumannii* does not display antioxidative activity. Neutrophils were stimulated with 200 nM PMA and co-cultured with *A. baumannii* (MOI 50) for 1 h. The expression of ROS in neutrophils was detected by flow cytometry using a total ROS detection kit (Enzo Life Sciences, Farmingdale, NY, USA). The mean fluorescence intensity was then measured and is shown. The data are shown as the mean ± SD; $n \geq 3$ per group; n.s., not significant. The results are representative of at least three experiments.

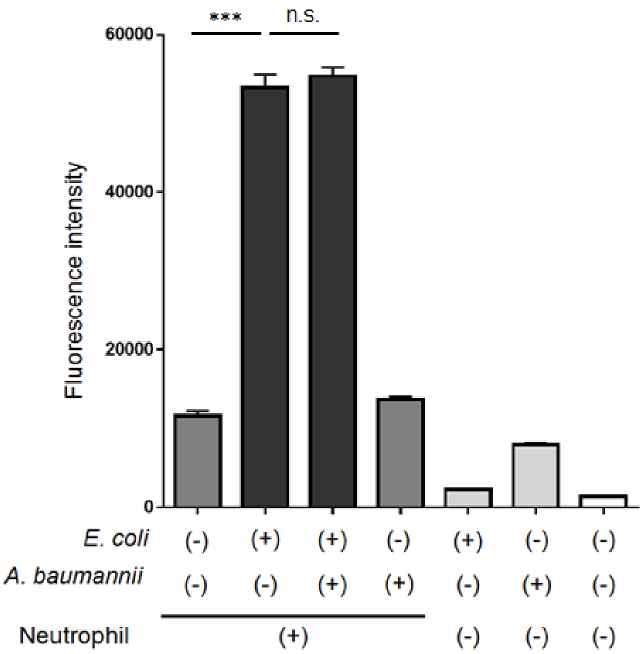

FIGURE S3 | *A. baumannii* does not inhibit bacterium-induced NET formation. Neutrophils and *E. coli* (MOI 50) were co-cultured for 3 h, in the presence or absence of *A. baumannii* (MOI 50). Extracellular DNA was stained with SYTOX green and the signal quantified. The data are shown as the mean ± SD; $n \geq 3$ per group; n.s., not significant; ***$p < 0.001$. The results are representative of at least three experiments.

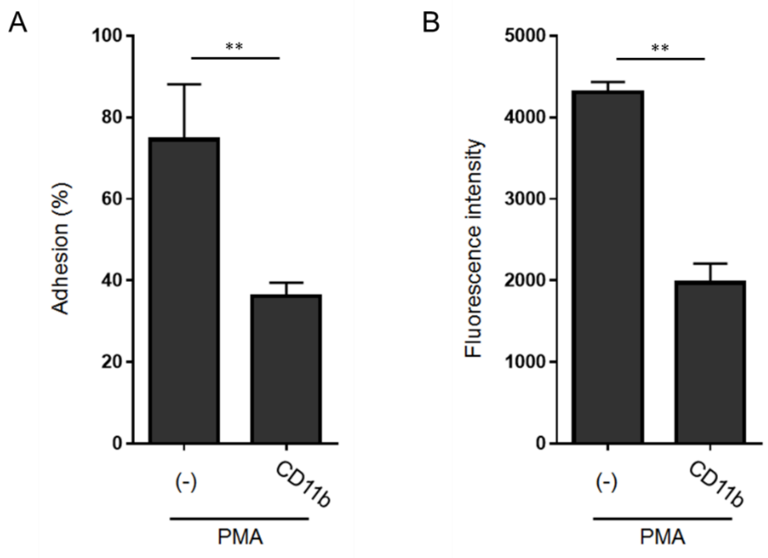

FIGURE S4 | Effect of the neutrophil cell surface molecule CD11b on cell adhesion and NET formation on PMA stimulation. **(A)** Neutrophils were stimulated with 200 nM PMA for 30 min, and the non-adherent neutrophils were removed; adherent cells were lysed, and LDH levels were determined. **(B)** Neutrophils were cultured for 3 h; the extracellular DNA was stained with SYTOX green, and the signal was quantified. In some experiments, the assay was performed in the presence of 0.1 μg/mL CD11b-blocking antibody. The data are shown as the mean ± SD; $n \geq 3$ per group. **p < 0.01. The results are representative of at least three experiments.

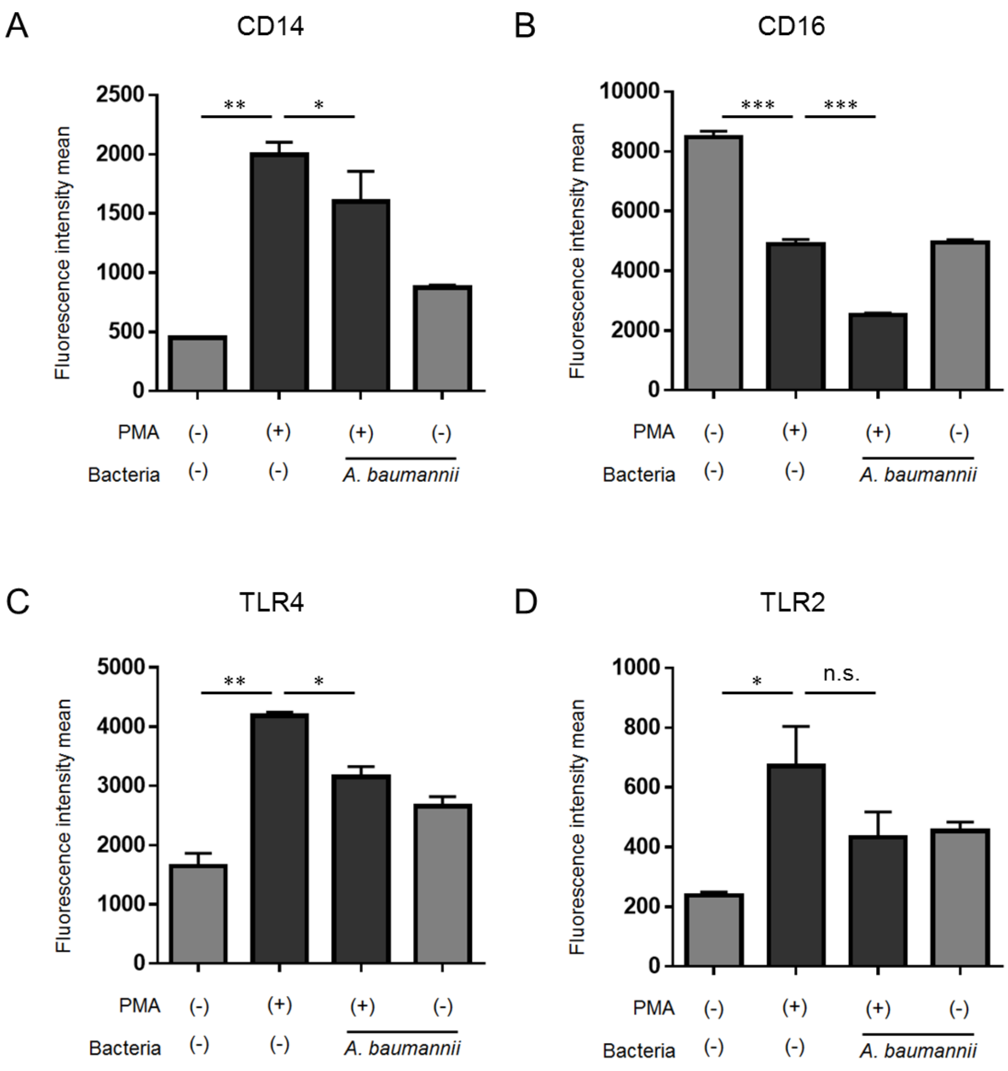

FIGURE S5 | Changes in cell surface expression of neutrophil molecules. Neutrophils were stimulated with 200 nM PMA and co-cultured with *A. baumannii* (MOI 50) for 1 h. The expression of CD14 **(A)**, CD16 **(B)**, TLR4 **(C)** and TLR2 **(D)** was determined by flow cytometry by using specific antibodies. The data are shown as the mean ± SD; $n \geq 3$ per group. n.s., not significant; ***p < 0.001, **$p < 0.01$, *$p < 0.05$. The results are representative of at least three experiments.

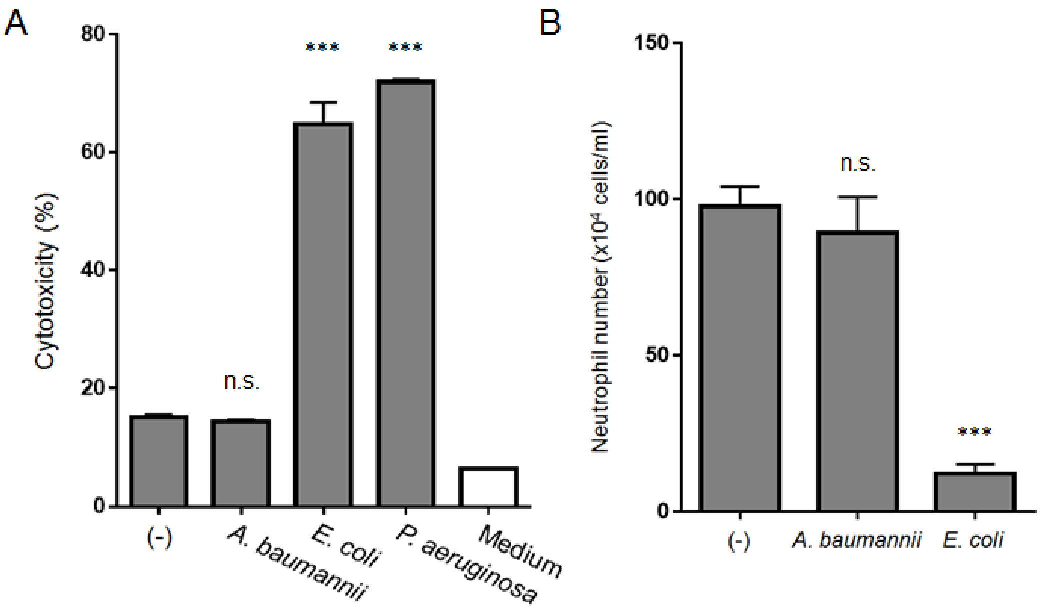

FIGURE S6 | *A. baumannii* does not kill neutrophils. **(A)** Neutrophils and *A. baumannii*, *E. coli*, or *P. aeruginosa* (PAO1 reference strain) (MOI 50) were co-cultured for 3 h. Extracellular LDH from neutrophils was detected by MTX-LDH assay kit. Cytotoxicity was calculated as a percentage compared with LDH levels of lysed neutrophils. **(B)** Neutrophils and *A. baumannii* or *E. coli* (MOI 50) were co-cultured for 3 h. The cells were stained with trypan blue, and the surviving cells were counted. The data are shown as the mean ± SD; $n \geq 3$ per group. n.s., not significant; ***$p < 0.001$ relative to culture in the absence of bacteria. The results are representative of at least three experiments.
